# 单形性嗜上皮性肠T细胞淋巴瘤的非典型临床病理特征

**DOI:** 10.3760/cma.j.cn121090-20241120-00459

**Published:** 2025-07

**Authors:** 丹婷 熊, 飞 程, 竞泽 许, 敬瀚 王, 亚飞 张, 艳艳 蔡, 文娟 干, 小秋 李, 照明 王, 芳 喻

**Affiliations:** 1 苏州大学附属第四医院病理科，苏州 215000 Department of Pathology, the Fourth Affiliated Hospital of Soochow University, Suzhou 215000, China; 2 浙江大学医学院附属第一医院病理科，杭州 310003 Department of Pathology, the First Affiliated Hospital, College of Medicine, Zhejiang University, Hangzhou 310003, China; 3 浙江大学医学院附属第一医院血液科，杭州 310003 Department of Hematology, the First Affiliated Hospital, College of Medicine, Zhejiang University, Hangzhou 310003, China; 4 浙江大学医学院附属第一医院PET中心，杭州 310003 PET center, the First Affiliated Hospital, College of Medicine, Zhejiang University, Hangzhou 310003, China; 5 复旦大学附属肿瘤医院病理科，上海 200032 Department of Pathology, Fudan University Shanghai Cancer Center, Shanghai 200032, China

**Keywords:** 淋巴瘤，T细胞, 诊断，鉴别, 免疫组织化学, Lymphoma, T-cell, Diagnosis, differential, Immunohistochemistry

## Abstract

**目的:**

探讨单形性嗜上皮性肠T细胞淋巴瘤（MEITL）的临床病理特征并讨论其诊断与鉴别诊断。

**方法:**

纳入2015年6月至2024年1月期间苏州大学附属第四医院和浙江大学医学院附属第一医院的MEITL病例共36例，进行免疫组化、EB病毒编码的小RNA（EBER）原位杂交和T细胞受体重排检测，收集临床、实验室检查及随访资料并进行相关性研究。

**结果:**

36例患者中男20例、女16例，中位年龄57（17～76）岁。22例（61％）出现肠道以外的病灶。32例（89％）接受手术和（或）化疗，1例接受auto-HSCT。中位随访时间为11.5（8～73）个月，34例（94％）死亡，中位生存期为6（1～67）个月。组织形态学结果显示，9例（25％）肿瘤细胞表现出显著的多形性，而非经典的单形性外观。免疫组织化学染色发现，P53和c-Myc高表达均与非典型组织形态学具有相关性（*P*＝0.003；*P*＝0.016）。高表达P53组的生存期短于低表达P53组（*χ*^2^＝4.922，*P*＝0.027），而c-Myc的表达水平对生存期无影响（*χ*^2^＝0.034，*P*＝0.854）。22例（68.8％）PD-L1联合阳性分数评分≥10分。EBER原位杂交检测显示，4例（11％）背景淋巴细胞呈阳性表达。17例（17/17，100.0％）检测到克隆性TCR基因重排。

**结论:**

MEITL是一种罕见的高度侵袭性淋巴瘤，部分病例可表现出非典型的临床及病理特征，提高对这类疾病的认识，有助于早期正确诊断及新治疗方案的确立。

## Abstract (en)

**Objective:**

This study sought to examine the clinicopathological features of monomorphic epitheliotropic intestinal T-cell lymphoma（MEITL）and to discuss its differential diagnosis.

**Methods:**

A total of 36 MEITL cases, collected between June 2015 and January 2024 from the Fourth Affiliated Hospital of Soochow University and the First Affiliated Hospital, College of Medicine, Zhejiang University, were analyzed. Patients underwent immunohistochemistry, in situ hybridization for Epstein-Barr virus-encoded small RNA（EBER）, and T-cell receptor（TCR）gene rearrangement testing. Clinical data, laboratory results, and follow-up information were collected for correlation analysis.

**Results:**

The cohort included 36 patients（20 males and 16 females）aged 17–76 years（median: 57 years）. Tumors outside the intestine were observed in 22 cases（61％）. A total of 32 patients（89％）underwent surgical intervention and/or chemotherapy, and one patient received auto-HSCT. The median follow-up duration was 11.5 months（range: 8–73 months）, with a median overall survival of 6 months（range: 1–67 months）; 34 patients died during the follow-up period. Morphologically, nine cases（25％）exhibited significant pleomorphism. Immunohistochemical analysis revealed that high expression levels of both P53 and c-Myc were correlated with atypical morphology（*P*＝0.003 and *P*＝0.016, respectively）. Notably, patients with high P53 expression had significantly shorter survival times than those with low P53 expression（*χ*^2^＝4.922, *P*＝0.027）, whereas survival did not differ significantly based on c-Myc expression levels（*χ*^2^＝0.034, *P*＝0.854）. Furthermore, a PD-L1 CPS score ≥10 was observed in 22 cases（68.8％）. Scattered EBER positivity in background cells was identified in four cases. All tested cases（17/17, 100.0％）showed clonal TCR gene rearrangements.

**Conclusion:**

MEITL is a rare but highly aggressive lymphoma with distinct clinical and pathological features. A subset of cases may exhibit atypical morphological patterns, complicating the diagnostic process. Improving awareness of this neoplasm is helpful for early and precise diagnosis as well as the estabolishment of novel therapy regimen.

单形性嗜上皮性肠T细胞淋巴瘤（monomorphic epitheliotropic intestinal T-cell lymphoma，MEITL）是一种罕见的原发性肠道T细胞淋巴瘤（primary intestinal T-cell lymphoma，PITL）[1]。在WHO的PITL分类中，MEITL已成为并列于肠病相关性T细胞淋巴瘤（EATL）的独立疾病实体[2]，但是如何与其他PITL鉴别仍然是诊断的难点。Hang等[3]研究认为伴有典型免疫表型但形态学出现多形性的病例应被归为MEITL的多形性变异型，而那些同时出现非典型形态学和非典型免疫表型的病例应被归为肠道T细胞淋巴瘤-非特指型（ITCL-NOS）。为了更好地认识这一罕见的疾病类型，本研究收集了36例MEITL病例，探讨其临床病理学特征，并分析其对预后的影响。

## 病例与方法

1. 病例资料：纳入2015年6月至2024年1月，在苏州大学附属第四医院病理科及浙江大学医学院附属第一医院病理科诊断为MEITL和Ⅱ型EATL的患者36例，所有病例均由2名淋巴瘤亚专科高年资医师确认，参照2022年第5版淋巴造血系统肿瘤WHO分类中MEITL的诊断标准[4]。患者临床病史及影像学资料均来自电子病历系统纪录。本研究经浙江大学医学院附属第一医院临床研究伦理委员会批准（批件号：浙大一院伦审2023研第0011号-快）。

2. 免疫组织化学染色：使用Ventana BenchMark XT全自动免疫组织化学染色仪（罗氏诊断产品有限公司，瑞士）进行Multimer法染色，检测抗体包括CD3、CD4、CD5、CD8、CD56、TIA-1、GranzymeB、P53、c-Myc、CD20、CD30、BCL2、MUM1、PD1、PD-L1（22C3）和Ki-67，均购自北京中杉金桥生物技术有限公司。P53染色模式判读分为高表达（≥50％肿瘤细胞核弥漫强着色）和低表达（<50％散在强弱不等的肿瘤细胞核着色），c-Myc的高表达阈值为40％。PD-L1（22C3）染色结果根据联合阳性分数（CPS）判读。

3. 原位杂交：使用EB病毒编码的小RNA（EBER）原位杂交检测试剂盒（购自北京中杉金桥生物技术有限公司），操作步骤按照试剂盒说明书进行，所有病例均设置阳性对照及阴性对照。

4. T细胞受体（TCR）克隆性检测：将DNA从经福尔马林固定、石蜡包埋的组织切片中提取出来，并按照BIOMED2步骤，使用聚合酶链反应（PCR）方法评估TRG、TRB和TRD。

5. 随访：随访信息来自与患者的电话沟通，随访截止日期为2024年9月30日，以生存时间（月）为预后指标。

6. 统计学处理：使用SPSS 29.0统计软件进行数据的统计学分析。计量资料使用*M*（范围）进行统计描述。计数资料以例（％）表示，组间比较采用Fisher精确检验，生存分析采用Kaplan-Meier法，生存率的比较采用Log-rank检验。*P*<0.05为差异具有统计学意义。

## 结果

1. 临床及影像学特征：36例MEITL患者中，男20例，女16例，中位年龄57（17～76）岁。21例（21/36，58.3％）出现急腹症伴有肠梗阻和（或）穿孔。所有病例均无乳糜泻病史。22例（22/36，66.1％）表现为肠道以外的病灶累及，包括肺（1例）、女性子宫附件（2例）、胆囊窝+胰腺（1例）、肾上腺（1例）、心包（1例）、淋巴结（15例）、骨髓（1例）。1例有急性T淋巴母细胞性淋巴瘤（T-LBL）病史。18例（18/36，50％）进行PET-CT检查，中位最大标准摄取值（SUVmax）为13.3（4.5～28.5）（图1A、B）。27例在手术切除后接受系统性化疗和（或）放疗，1例在auto-HSCT后复发，4例仅进行手术切除，4例在活检确诊后进行系统性化疗。

2. 组织形态学及免疫组化特征：①大体检查：32例手术切除标本中，中位肿瘤长径为8.0（2.7～17.5）cm，肠壁全层受累。②镜下观察：27例（27/36，75.0％）出现典型的MEITL形态学特征，即小至中等大的肿瘤细胞具有显著的单形性特征。9例（9/36，25.0％）肿瘤细胞呈多形性，表现为肿瘤细胞体积增大，伴有空泡状染色质和显著的核仁（图1C），其中1例（1/9，11.1％）观察到明显的血管侵犯（图1D），6例（6/9，66.7％）出现凝固性坏死及凋亡。免疫组织化学染色检测发现，所有病例均显示CD3弥漫强阳性表达和CD4阴性表达，所有病例均不同程度表达细胞毒性标志物（TIA-1 100％、granzyme B 87.0％）。除33例（33/36，91.7％）显示典型的CD8^+^/CD56^+^表达外，2例（2/36，5.6％）显示CD8^+^/CD56^-^，1例（1/36，2.8％）显示CD8^-^/CD56^+^。10例（10/34，29.4％）显示P53高表达（图1E），P53高表达与非典型组织形态学具有相关性（*P*＝0.003）。10例（10/33，30.3％）显示c-Myc高表达（图1F），c-Myc高表达与非典型组织形态学具有相关性（*P*＝0.016）。P53、c-Myc与非典型组织形态学的相关性分析见图2A。32例进行PD-L1免疫组织化学染色，其中22例（22/32，68.8％）PD-L1 CPS评分≥10分，范围10～70分（图1G）。所有病例均显示较高的Ki-67增殖指数，中位数为80％（50％～95％）。

**图1:**
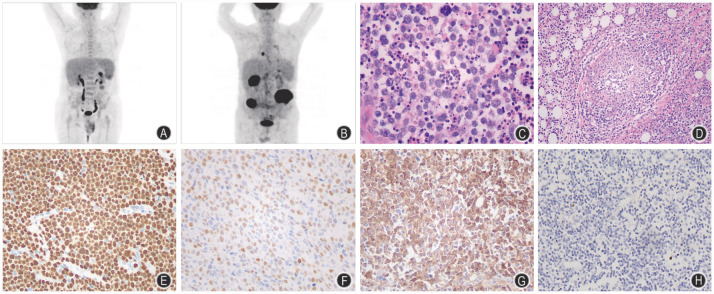
单形性嗜上皮性肠T细胞淋巴瘤影像学及病理学特征 **A** PET-CT检查示左侧中腹部局部肠壁似略增厚，SUVmax为4.5；**B** PET-CT检查示回盲部及降结肠肠壁、胆囊窝处团块状软组织密度影，胰尾部结节样^18^F-氟代脱氧葡萄糖代谢增高，SUVmax为28.5；**C** 肿瘤细胞核大、异形明显伴广泛的凋亡和组织细胞吞噬（HE染色，×400）；**D** 肿瘤细胞侵犯血管壁（HE染色，×100）；**E** 免疫组织化学染色显示P53弥漫强阳性表达（Multimer，×200）；**F** 免疫组织化学染色显示c-Myc高表达（Multimer，×200）；**G** 免疫组织化学染色显示PD-L1联合阳性分数（CPS）评分为70分（Multimer，×200）；**H** EB病毒编码的小RNA原位杂交检测显示个别背景细胞阳性（×200）

3. EBER原位杂交和TCR重排检测：所有病例均进行EBER原位杂交检测，4例（4/36，11.1％）显示个别反应性淋巴细胞阳性（图1H）。17例（17/17，100％）检测到克隆性TCR基因重排。

4. 生存分析：中位随访时间为11.5（8～73）个月，34例死于疾病，中位生存期为6（1～67）个月。截至末次随访，有2例存活，生存时间分别为8个月和30个月。与P53低表达组比较，P53高表达组的生存期明显缩短（*χ*^2^＝4.922，*P*＝0.027；图2B）。c-Myc低表达组与c-Myc高表达组生存期差异无统计学意义（*χ*^2^＝0.034，*P*＝0.854；图2C）。

**图2:**
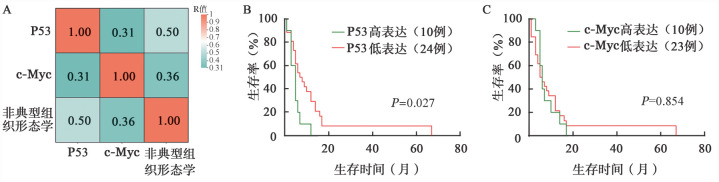
P53、c-Myc与非典型组织形态学的相关性分析及对单形性嗜上皮性肠T细胞淋巴瘤（MEITL）患者生存的影响 **A** P53、c-Myc表达与非典型组织形态学的相关性分析；**B** P53的表达对MEITL患者生存的影响；**C** c-Myc的表达对MEITL患者生存的影响

## 讨论

MEITL是一种侵袭性PITL，其特征是单形性细胞形态学和亲上皮性，通常不与乳糜泻相关[4]–[7]。MEITL在全球范围内均有分布，在亚洲，MEITL病例的数量远远多于EATL[8]。为了更好地认识这一罕见的疾病类型，我们收集2家单位共计36例MEITL病例，探讨其临床病理学特征及预后分析。

36例MEITL病例中，男性居多（男女比例为5∶4），大多为中老年人，40岁以下2例（例11为17岁；例30为35岁）。例11因为发病年龄不典型，且存在T-LBL病史，直到依据肠穿孔的手术切除标本才得以正确诊断。MEITL的病理形态学特征表现为相对单一的中等大小淋巴瘤细胞密集浸润，常见亲肠上皮现象，但缺乏炎症背景。本研究中，部分病例观察到异形性明显的肿瘤细胞浸润，出现空泡状染色质以及显著的核仁。还观察到“星空现象”、坏死以及亲血管现象。这些不典型的形态学改变，容易与非特指外周T细胞淋巴瘤和NK/T细胞淋巴瘤混淆，但由于肿瘤细胞特征性表达CD5^-^、CD4^-^、CD8^+^、CD56^+^、EBER^-^，仍然诊断为MEITL。随后的免疫组织化学染色发现，这些形态不典型的病例，大多高表达P53和c-Myc蛋白，肯德尔（kendall）相关系数分别为0.5和0.36。同时存在少数免疫组化表达模式不典型的病例，2例显示CD8^+^/CD56^-^、1例显示CD8^-^/CD56^+^，但未出现CD4^+^或CD8^-^/CD56^-^的病例。

MEITL多局限于胃肠道，可出现消化道外累及，例如肺、皮肤和中枢神经系统[9]–[12]。本研究中除消化道受累以外，其他受累脏器有肺、心包、胆囊、胰腺、肾上腺、女性子宫附件、淋巴结和骨髓。如果忽略共存的胃肠道病灶，则可能误诊为其他T细胞淋巴瘤。在18例进行PET-CT检查的病例中，7例SUVmax低于10，描述为肠壁增厚伴放射性摄取增高，与炎性病变难以鉴别。近年来随着单/双球囊技术的发展，能观察到远端十二指肠、空肠和大部分回肠，有助于早期发现MEITL[13]。有研究显示MEITL较特异的早期内镜下特点表现为水肿性和微红色病变（颗粒状黏膜）[14]。本研究中，有4例通过活检样本确诊，但均表现为明确的肿块。活检样本判读时需要鉴别另外2个发生在胃肠道的惰性淋巴瘤/增生性疾病：胃肠道惰性T细胞淋巴瘤和胃肠道惰性NK细胞淋巴增殖性疾病。二者均可出现轻度异形的肿瘤细胞表达CD8^+^/CD56^+^，但前者Ki-67增殖指数<10％[15]–[17]；后者肿瘤细胞胞质嗜酸性，且内镜检查显示为单个或多个浅表小病变，表现为息肉样隆起[18]–[20]。

MEITL的治疗方法主要包括手术切除、辅助性放化疗以及auto-HSCT，但总体均无法显著改善患者预后，可能与MEITL未被及早发现，常因肠穿孔就诊有关[6],[21]。有研究显示，早期发现的MEITL患者在首次诊断后使用CHOP（环磷酰胺、阿霉素、长春新碱和泼尼松）方案治疗出现68个月的完全缓解，提示早期发现可能会改善预后，这也需要内镜医师特别注意到MEITL的早期内镜下改变[21]。本研究27例患者手术切除后接受系统性化疗和（或）放疗，1例行auto-HSCT后复发。例20在初发手术切除后，仅进行了1个疗程化疗，缓解5年后复发，总体生存期达67个月，推测早期单一病灶的根治性切除是影响预后的决定性因素。我们发现P53高表达患者的生存期明显缩短。我们还对本组患者回顾性地进行PD-L1表达研究，显示22例CPS评分≥10分，但无患者接受免疫治疗。本组患者预后差，截至末次随访，仅2例患者存活，目前难以在大样本中明确免疫治疗与MEITL预后改善是否存在相关性，因此MEITL患者能否从免疫治疗中获益还需进一步的研究证实。

综上，本研究总结了MEITL不典型的临床表现和病理形态学及免疫组化表达特征，这些不典型病例扩宽了MEITL的临床病理学谱系，同时也为MEITL的诊断和治疗带来了新的挑战。

## References

[1] Isaacson PG, Chott A, Ott G (2008). Enteropathy-associated T-cell lymphoma[M]//WHO Classification of Tumours of Haematopoietic and Lymphoid Tissues.

[2] Jaffe ES, Chott A, Ott G (2017). Monomorphic epitheliotropic intestinal T-cell lymphoma[M]//WHO Classification of Tumours of Haematopoietic and Lymphoid Tissues.

[3] Hang JF, Yuan CT, Chang KC (2022). Targeted next-generation sequencing reveals a wide morphologic and immunophenotypic spectrum of monomorphic epitheliotropic intestinal t-cell lymphoma[J]. Am J Surg Pathol.

[4] Miranda RN, Amador C, Chan J (2024). Fifth Edition of the World Health Organization classification of tumors of the hematopoietic and lymphoid tissues: mature t-cell, nk-cell, and stroma-derived neoplasms of lymphoid tissues[J]. Mod Pathol.

[5] Chott A, Haedicke W, Mosberger I (1998). Most CD56+ intestinal lymphomas are CD8+CD5-T-cell lymphomas of monomorphic small to medium size histology[J]. Am J Pathol.

[6] Veloza L, Cavalieri D, Missiaglia E (2023). Monomorphic epitheliotropic intestinal T-cell lymphoma comprises morphologic and genomic heterogeneity impacting outcome[J]. Haematologica.

[7] Chan JK, Chan AC, Cheuk W (2011). Type II enteropathy-associated T-cell lymphoma: a distinct aggressive lymphoma with frequent γδ T-cell receptor expression[J]. Am J Surg Pathol.

[8] Yi JH, Lee GW, Do YR (2019). Multicenter retrospective analysis of the clinicopathologic features of monomorphic epitheliotropic intestinal T-cell lymphoma[J]. Ann Hematol.

[9] Soardo G, Castaldo V, Donnini D (2020). Monomorphic Epitheliotropic Intestinal T Cell Lymphoma of the Appendix: a Case Report and Review of Literature[J]. J Gastrointest Cancer.

[10] Olmos-Alpiste F, Vázquez I, Gallardo F (2021). Monomorphic epitheliotropic intestinal T-Cell lymphoma with secondary cutaneous involvement: a diagnostic challenge[J]. Am J Dermatopathol.

[11] Suzuki Y, Minemura H, Tomita H (2020). Monomorphic epitheliotropic intestinal t-cell lymphoma involving the lung and brain: a rare case study[J]. Intern Med.

[12] Morimoto A, Fujioka Y, Ushiku T (2021). Monomorphic epitheliotropic intestinal t-cell lymphoma invades brain[J]. Intern Med.

[13] Naito T, Nosaka T, Takahashi K (2022). Successful early diagnosis of monomorphic epitheliotropic intestinal T-cell lymphoma manifesting as chronic diarrhea and hypokalemia using video capsule endoscopy and double-balloon enteroscopy[J]. Clin J Gastroenterol.

[14] Aoki Y, Sujino T, Takabayashi K (2021). Various Endoscopic features in monomorphic epitheliotropic intestinal T-Cell lymphoma[J]. Case Rep Gastroenterol.

[15] Tan SY, Chuang SS, Tang T (2013). Type II EATL (epitheliotropic intestinal T-cell lymphoma): a neoplasm of intra-epithelial T-cells with predominant CD8αα phenotype[J]. Leukemia.

[16] Küçük C, Wei L, You H (2020). Indolent T-Cell Lymphoproliferative disease of the GI tract: insights for better diagnosis, prognosis, and appropriate therapy[J]. Front Oncol.

[17] de Leval L, Feldman AL, Pileri S (2023). Extranodal T- and NK-cell lymphomas[J]. Virchows Arch.

[18] Matnani R, Ganapathi KA, Lewis SK (2017). Indolent T- and NK-cell lymphoproliferative disorders of the gastrointestinal tract: a review and update[J]. Hematol Oncol.

[19] Attieh M, Asakrah S (2023). Indolent NK-cell lymphoproliferative disorder of the GI tract: a rare indolent neoplasm of the GI tract[J]. Blood.

[20] Soderquist CR, Bhagat G (2020). Gastrointestinal T- and NK-cell lymphomas and indolent lymphoproliferative disorders[J]. Semin Diagn Pathol.

[21] Ozaka S, Inoue K, Okajima T (2021). Monomorphic epitheliotropic intestinal T-cell lymphoma presenting as melena with long-term survival: a case report and review of literature[J]. World J Gastroenterol.

